# Predictive value of liver and spleen stiffness measurement based on two-dimensional shear wave elastography for the portal vein pressure in patients with compensatory viral cirrhosis

**DOI:** 10.7717/peerj.15956

**Published:** 2023-09-15

**Authors:** Peng Wang, Xinhong Hu, Feng Xie

**Affiliations:** Functional Department, The No.2 People’s Hospital of Lanzhou, Lanzhou, China

**Keywords:** Compensatory viral cirrhosis, Two-dimensional shear-wave elastic imaging, Liver and spleen stiffness, Portal vein pressure, Correlation, Predictive value

## Abstract

**Objective:**

This study aimed to explore the predictive value of liver and spleen stiffness measurement based on two-dimensional shear wave elastography for the portal vein pressure in patients with compensatory viral cirrhosis.

**Methods:**

From January 2017 to August 2019, 107 patients with compensatory viral cirrhosis and 76 patients with viral hepatitis were enrolled as cirrhosis group and hepatitis group, respectively. Patient data were obtained during admission, and this study was a review and analysis of patient data. Liver stiffness measurement (LSM), spleen stiffness measurement (SSM), portal vein diameter and spleen thickness were compared between the two groups, and their diagnostic value for compensatory viral cirrhosis was analyzed. According to the hepatic vein pressure, the cirrhosis group patients were divided into non-hypertensive group (no portal hypertension, hepatic venous pressure gradient (HVPG) < 5 mmHg), mild group (mild portal hypertension, 5 mmHg ≤ HVPG ≤ 10 mmHg) and severe group (clinically significant portal hypertension group, HVPG > 10 mmHg). LSM, SSM, portal vein diameter and spleen thickness of the three groups were compared, and the correlation between SSM and hepatic vein pressure was analyzed.

**Results:**

LSM, SSM, portal vein diameter and spleen thickness in the cirrhosis group were higher than those in hepatitis group (all *P* < 0.05). The area under the curve (AUC) of combined detection was larger than that of LSM, SSM and spleen thickness detection alone in liver cirrhosis diagnosis (all *P* < 0.05). LSM, SSM, portal vein diameter and spleen thickness increased with the increase of hepatic vein pressure in patients with liver cirrhosis (all *P* < 0.05). LSM, SSM, portal vein diameter and spleen thickness were all positively correlated with hepatic vein pressure (*P* < 0.05). ROC curve showed that AUC of combined detection was greater than that of LSM, SSM, portal vein diameter and spleen thickness alone detection in the diagnosis of clinically significant portal hypertension (all *P* < 0.05). The increase of LSM, SSM, portal vein diameter and spleen thickness were the influencing factors for hepatic vein pressure rising (all *P* < 0.05).

**Conclusion:**

There was an increase of LSM and SSM in patients with compensatory viral cirrhosis, which were positively correlated with hepatic venous pressure, and combined index detection has diagnostic and predictive value for the change of portal venous pressure.

## Introduction

Many liver diseases develop liver cirrhosis in their advanced stages. In the compensatory stage liver cirrhosis has no typical clinical manifestations; however, in the decompensated stage, it can be manifested as ascites, esophageal and gastric variceal bleeding and other complications. Portal hypertension is the main complication of liver cirrhosis. Most of the normal liver blood supply comes from the portal vein. The anatomical structure of the portal vein is relatively special, with its the beginning and end are capillaries. When the degree of liver cirrhosis is aggravated, the blood vessels in the liver parenchyma gradually become thinner, and the pressure of the portal vein increases, forcing much blood to flow reversely and enter the collateral circulation, resulting in esophageal and gastric varices, which affects the prognosis of patients (Liang et al., 2021; Zheng et al., 2021). The degree of portal hypertension is closely related to the severity of liver disease. Detection of portal hypertension can assess the severity of cirrhosis and provide timely and effective diagnosis for patients with liver disease. At present, the measurement of hepatic vein pressure gradient is often used to assess the severity of portal hypertension, but it is an innovative operation with high limitations. Ultrasonic elastography is an inspection technology developed based on two-dimensional ultrasound. It uses the probe to compress the tissue longitudinally to generate the longitudinal strain force inside the tissue. The uneven elastic distribution the tissue will generate different strain force, so the situation inside the tissue can be evaluated (Yuli et al., 2020; Zhilin et al., 2021). Two-dimensional shear wave elastography is the latest elastography technology. It estimates the tissue hardness by measuring the propagation velocity of elastic shear wave in the tissue. Compared with ultrasonic elastography, it has evident advantages: it can perform elastography based on two-dimensional images; it does not need to apply pressure during operation; it can observe the two-dimensional images in real time with avoiding the pipeline structure in the tissue; and it can measure the elastic modulus value in full quantity. Thus, it can be used to judge the degree of tissue lesions (Garteiser et al., 2021; Zhang et al., 2020). Based on this, this study aimed to explore the predictive significance of liver and spleen stiffness measurement based on two-dimensional shear wave elastography for the changes of portal vein pressure in patients with compensatory viral cirrhosis, and to provide a reference basis for the development of assessment methods of portal vein pressure changes.

## Materials & Methods

### Clinical data

From January 2017 to August 2019, 107 patients with compensatory viral cirrhosis and 76 patients with viral hepatitis were taken as cirrhosis group and hepatitis group, respectively. There were no significant differences in general data between the two groups (all *P* > 0.05, Table 1). The study was approved by the Institutional Review Board and Research Ethics Committee of the No.2 People’s Hospital of Lanzhou and was conducted in accordance with the tenets of the Declaration of Helsinki and the Ethics Committee of No.2 People’s Hospital of Lanzhou agreed to waive the informed consent.

**Table 1:** Comparison of general data between cirrhosis group and hepatitis group.

General data	Cirrhosis group (*n* = 107)	Hepatitis group (*n* = 76)	*χ* ^2^ */t*	*P*
Gender (cases)			0.625	0.429
Male	64	41		
Female	43	35		
Age (years)	53.52 ± 5.16	54.87 ± 5.59	1.685	0.094
Course of disease (years)	4.09 ± 0.63	3.98 ± 0.61	1.179	0.240
BMI (kg/m^2^)	23.18 ± 2.52	23.51 ± 2.73	0.843	0.400
Complicated underlying diseases				
Hyperlipidemia	31	18	0.634	0.426
Hypertension	19	12	0.122	0.727
Coronary heart disease	10	5	0.452	0.501

### Inclusion criteria

Patients (1) who met the diagnostic criteria of chronic viral hepatitis (Guang & Qun, 2010) and the diagnostic criteria for liver cirrhosis in the Expert Consensus on the Diagnosis and Treatment of Esophageal and Fundus Varices Rupture in the Cirrhotic portal Hypertension (2019 edition) (Spleen Portal Hypertension Surgery Group of Surgery Branch of Chinese Medical Association, 2019); (2) with complete clinical data; and (3) received two-dimensional shear wave elastography examination.

### Exclusion criteria

(1) Patients with cirrhosis caused by other reasons; (2) patients complicated with hepatocellular carcinoma, portal vein thrombosis and other serious complications; (3) patients with splenic lesions; (4) patients complicated with blood diseases; (5) patients with connective tissue diseases; and (6) patients with active infectious diseases.

### Methods

#### Two-dimensional shear-wave elastography examination

Two-dimensional shear wave belongs to the shear wave velocity imaging technology. Plane shear wave is the multi-point focused acoustic radiation force pulse generated by a common ultrasonic probe. The ultra-high-speed image processing technology can obtain two-dimensional shear wave elastic map, which can reflect the absolute hardness of the liver. Real-time shear-wave elastography of liver was performed on all patients using supersonic imagine aixplorer machine and SC6-1 abdominal probe. The patient was placed in a supine position, and his right upper limb was raised and placed on his head, fully exposing the right abdomen. Two-dimensional ultrasonography was performed from the right axillary front to the midaxillary line between the 4th and 7th ribs. The coupling agent was evenly applied on the skin surface with avoiding the thick pipe structures such as hepatic blood vessels and bile ducts and the location of gallbladder. The probe was vertical to the skin. After the 2D image was clearly displayed, the machine was switched to SWE mode. The sampling frame range was 4cm*3cm, the area of interest was set to 20.0 mm, and the elastic measurement SCALE was set to 40 kPa. Liver parenchyma about 2 cm below the capsule of the right lobe of liver was selected as the upper edge. Patients were asked to hold their breath for 3 to 5 s in a calm state, and the measurement was considered successful if the color of the sampling box was more than 90% full. The region with relatively uniform image color was selected for detection, and the average value of elastic modulus in the detection region was displayed. It was detected for 5 times in total and the average value was taken as LSM. For SSM detection, the patient was placed in the right decubitus position, with the left arm fully extended up and placed on the top of the head. The left midline or the posterior axillary line between the 9th and 11th ribs was selected. The probe was placed in the thickest middle part of the spleen, and the sampling range was set to 4 cm*3 cm. The spleen parenchyma about 1 cm below the capsule was selected as the upper margin, and the area of interest was 20.0 mm. The elastic measurement SCALE was 70 kPa. The measurement method was the same as that of LSM (Paisant et al., 2022).

#### Portal vein diameter and spleen thickness examination

Philips EPIQ7 and Mindray M5 color Doppler ultrasound diagnostic instrument with probe frequency of 3–9 MHz were applied. The patient was placed in supine position with his hands raised above his head. When scanning was not satisfactory, the position of the patient could be changed appropriately, and his breathing should be maintained steadily. The longitudinal section of the first porta hepatis under the right costal margin was taken as the standard measurement section. The examination showed that the common bile duct was full length and posterior to the head of pancreas. The portal vein was measured at 1∼2 cm from the first hilum. The splenic hilum and splenic vein were shown by scanning along the intercostal oblique section. The diameter line from the hilum to the opposite side of the spleen was measured as the thickness of the spleen (Elkerdawy et al., 2021).

#### Hepatic vein pressure gradient detection

Hepatic venous pressure gradient refers to the measurement of hepatic venous wedge pressure and free pressure by jugular catheter. Before examination, the necessity and risks of hepatic venous pressure testing should be explained to patients and their families. The testing can be carried out only after signing the informed letter of interventional diagnosis and treatment. The patient was instructed to take supine position and turn his head to the opposite side of puncture as far as possible. The right internal jugular vein was selected as puncture point, and local anesthesia was performed with 2% lidocaine. After successful puncture of the right internal jugular vein with the Seldinger technique, the 5F venipecture sheath (TERUMO, Tokyo, Japan) was inserted with the guide wire. The Cordis 5F multifunctional angiography catheter (Cordis, Hialeah, FL, USA) was inserted into the main right hepatic vein under X-ray fluoroscopy. After successful introduction was confirmed by angiography by infusion of 2 mL iohexol, the automatic manometer was connected to read the pressure data, namely, the free hepatic venous pressure (FHVP). Guided by the guide wire, the multifunctional catheter was continued to reach the end of the hepatic vein, and 2 mL iohexol was injected to confirm the angiography. The pressure data at this time was read as the wedged hepatic venous pressure (WHVP). The difference of the two was the hepatic venous pressure gradient (HVPG). Based on the hepatic venous pressure, the cirrhosis group was divided into the non-hypertension group (no portal hypertension, HVPG <5 mmHg), the mild group (mild portal hypertension group, 5 mmHg ≤ HVPG ≤ 10 mmHg), and the severe group (clinically significant portal hypertension group, HVPG > 10 mmHg) (Lesmana, Raharjo & Gani, 2020).

### Observation Indicators: baseline data, LSM, SSM, portal vein diameter and spleen thickness of patients with cirrhosis and hepatitis were collected

(1) LSM, SSM, portal vein diameter and spleen thickness were compared between the cirrhosis group and the hepatitis group, and their diagnostic value for compensatory viral cirrhosis was analyzed. (2) LSM, SSM, portal vein diameter and spleen thickness of the non-hypertension group, mild group and severe group were compared, and the correlation between each index and hepatic vein pressure, as well as the diagnostic value of clinically significant portal vein hypertension, was analyzed.

### Statistical analysis

SPSS22.0 software was used to process the data. The categorical data were expressed as %, and the difference between groups was compared by *χ*^2^ test. The measurement data were expressed by ($\bar {x}~\pm $ S) after normal test and compared by *t* test. ROC curve was used to analyze the diagnostic value of LSM, SSM, portal vein diameter and spleen thickness for compensatory viral cirrhosis. Spearman test was applied to analyze the correlation of LSM, SSM, portal vein diameter and spleen thickness with hepatic vein pressure. Logistic regression was used to analyze the correlation of LSM, SSM, portal vein diameter and spleen thickness with clinically significant portal hypertension. *P* < 0.05 meant that the difference was statistically significant.

## Results

### Comparison of LSM, SSM, portal vein diameter and spleen thickness between cirrhosis group and hepatitis group

**Figure 1:**
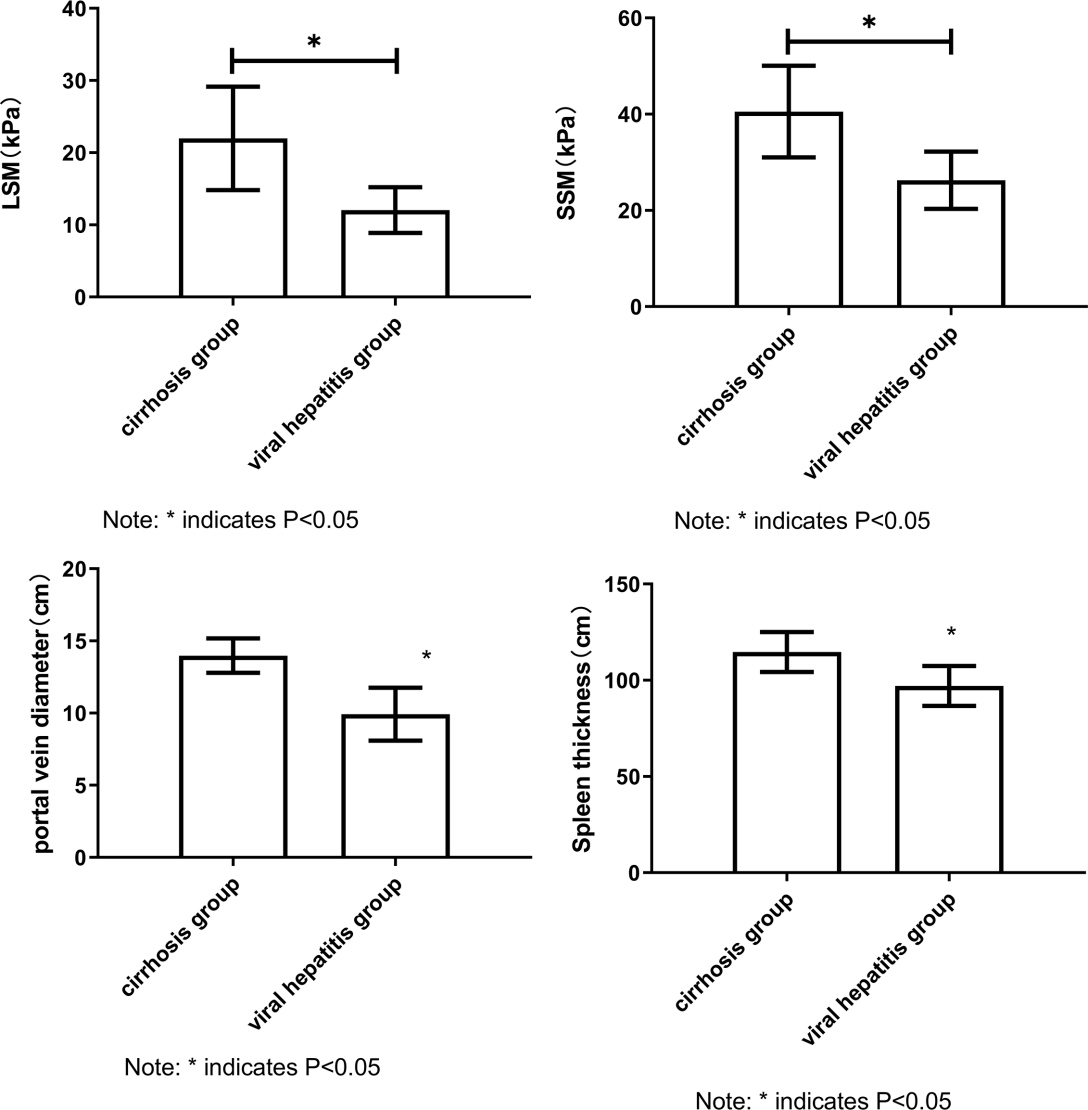
Comparison of LSM, SSM, portal vein diameter and spleen thickness between cirrhosis group and hepatitis group. Note: Compared with the cirrhosis group, * *P* < 0.05.

The values of LSM, SSM, portal diameter and spleen thickness in cirrhosis group were all higher than those in hepatitis group. The results showed that LSM, SSM, portal vein diameter and spleen thickness were correlated with the severity of liver disease, which provided a basis for further correlation analysis (all *P* < 0.05, Fig. 1) (Independent sample t test).

### Diagnostic value of LSM, SSM, portal vein diameter and spleen thickness for cirrhosis

**Figure 2:**
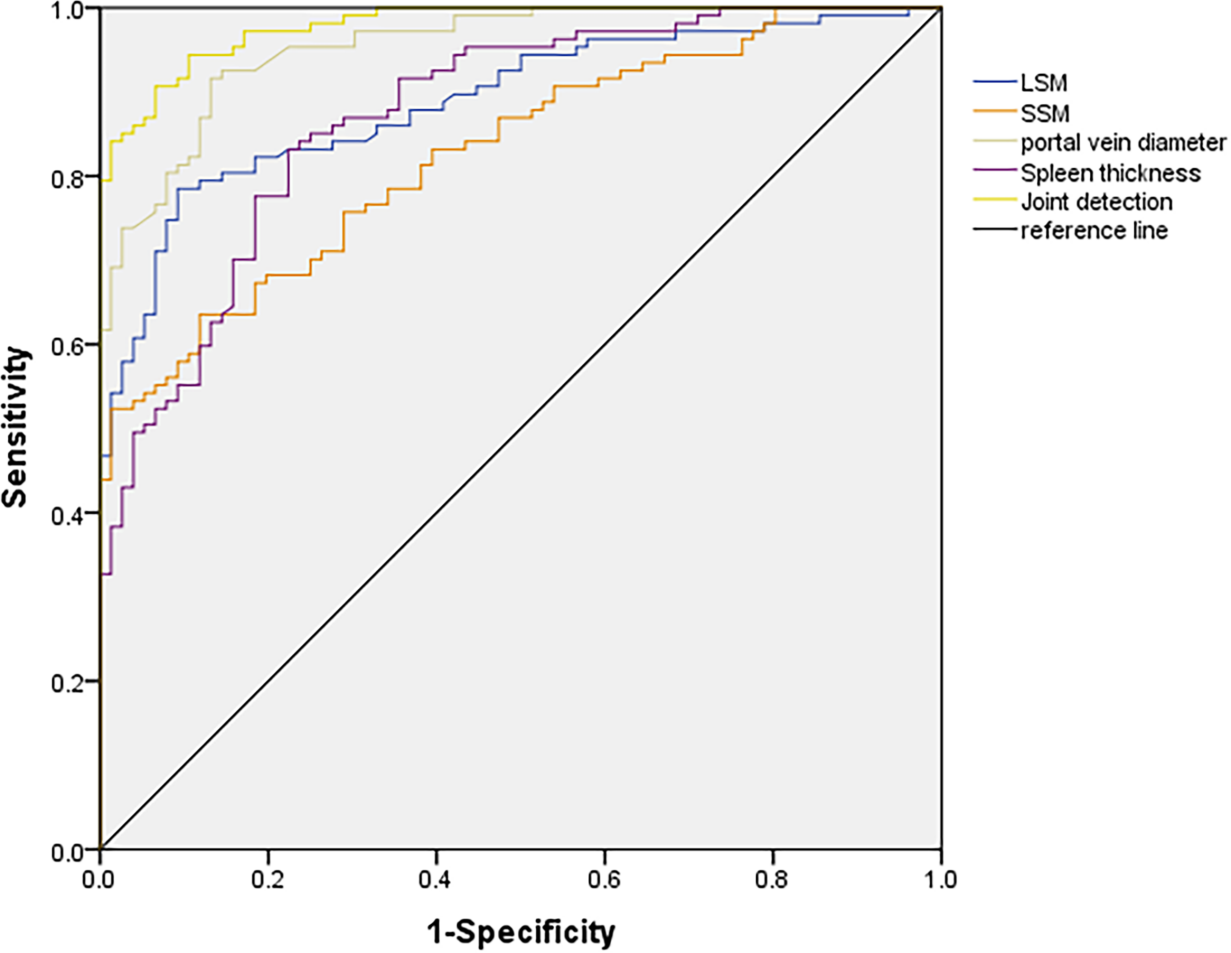
ROC curves of LSM, SSM, portal vein diameter and spleen thickness in differentiating hepatitis and cirrhosis.

The AUC of combined detection was greater than that of LSM, SSM and spleen thickness detection alone. The results showed that the combined detection of LSM, SSM, portal vein diameter and spleen thickness was effective, and the combined detection could be used to diagnose cirrhosis (all *P* < 0.05, Table 2 and Fig. 2) (Receiver operating characteristic curve (ROC) test).

**Table 2:** Diagnostic value of LSM, SSM, portal vein diameter and spleen thickness for cirrhosis.

Indicator	Cutoff value	AUC	SE	95% CI
LSM	16.52kPa	0.889^*^	0.024	0.843∼0.935
SSM	35.59kPa	0.827^*^	0.029	0.770∼0.884
Portal vein diameter	11.24cm	0.954	0.013	0.928∼0.980
Spleen thickness	109.37cm	0.872^*^	0.026	0.822∼0.922
Combination		0.979	0.008	0.964∼0.994

**Notes.**

Note: Compared with combination, * *P* < 0.05.

### Comparison of LSM, SSM, portal diameter and spleen thickness in patients with different hepatic venous pressures

**Figure 3:**
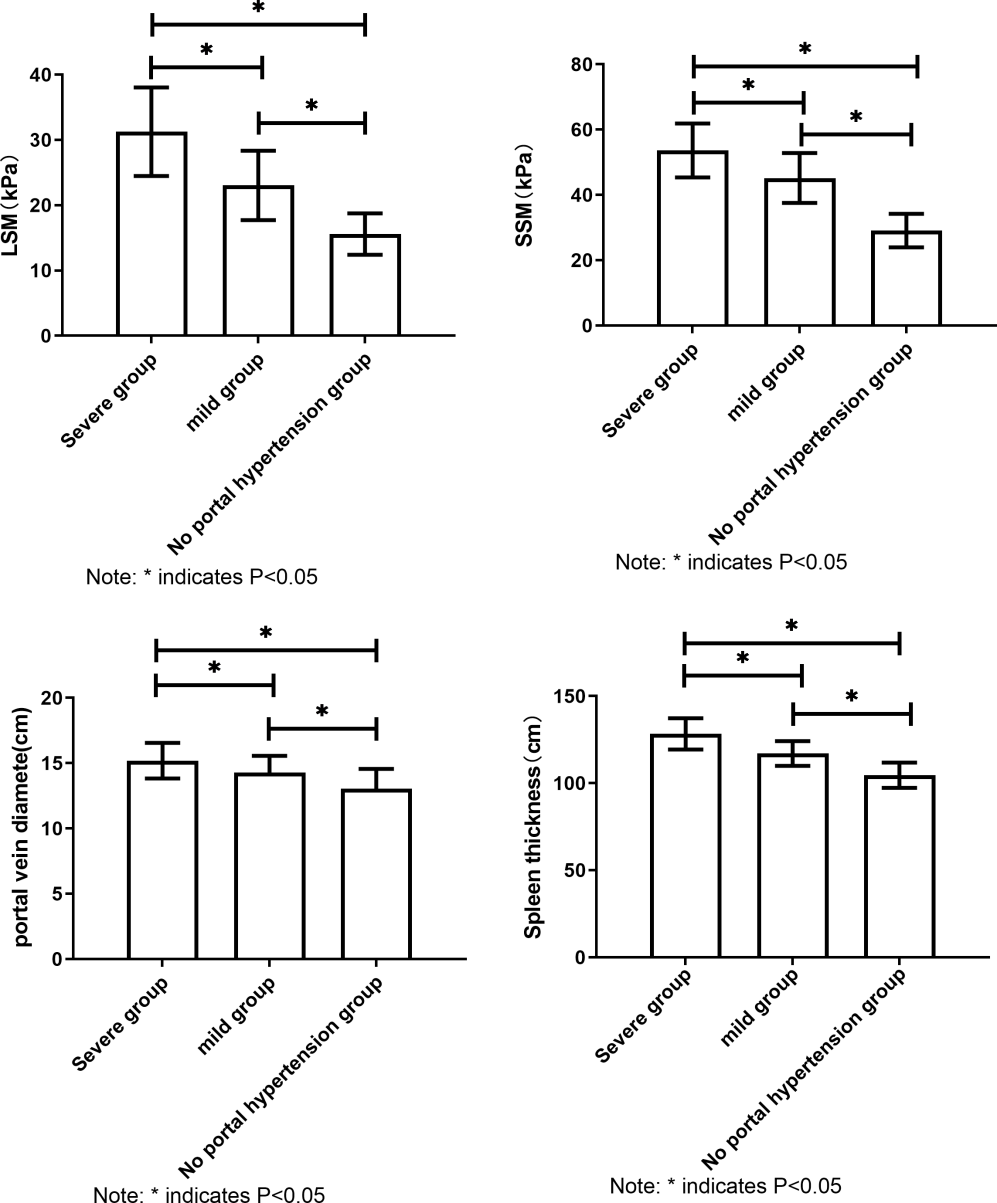
Comparison of LSM, SSM, portal vein diameter and spleen thickness in patients with different hepatic vein pressures. Note: All the comparisons of LSM, SSM, portal vein diameter and spleen thickness in patients with different hepatic venous pressure showed *P* < 0.05.

LSM, SSM, portal vein diameter and spleen thickness increased with the increase of hepatic vein pressure. The results showed that LSM, SSM, portal vein diameter and spleen thickness were correlated with hepatic venous pressure in cirrhotic patients (all *P* < 0.05, Fig. 3) (one-way analysis of variance).

### Correlation analysis of LSM, SSM, portal vein diameter and spleen thickness with hepatic vein pressure

**Figure 4:**
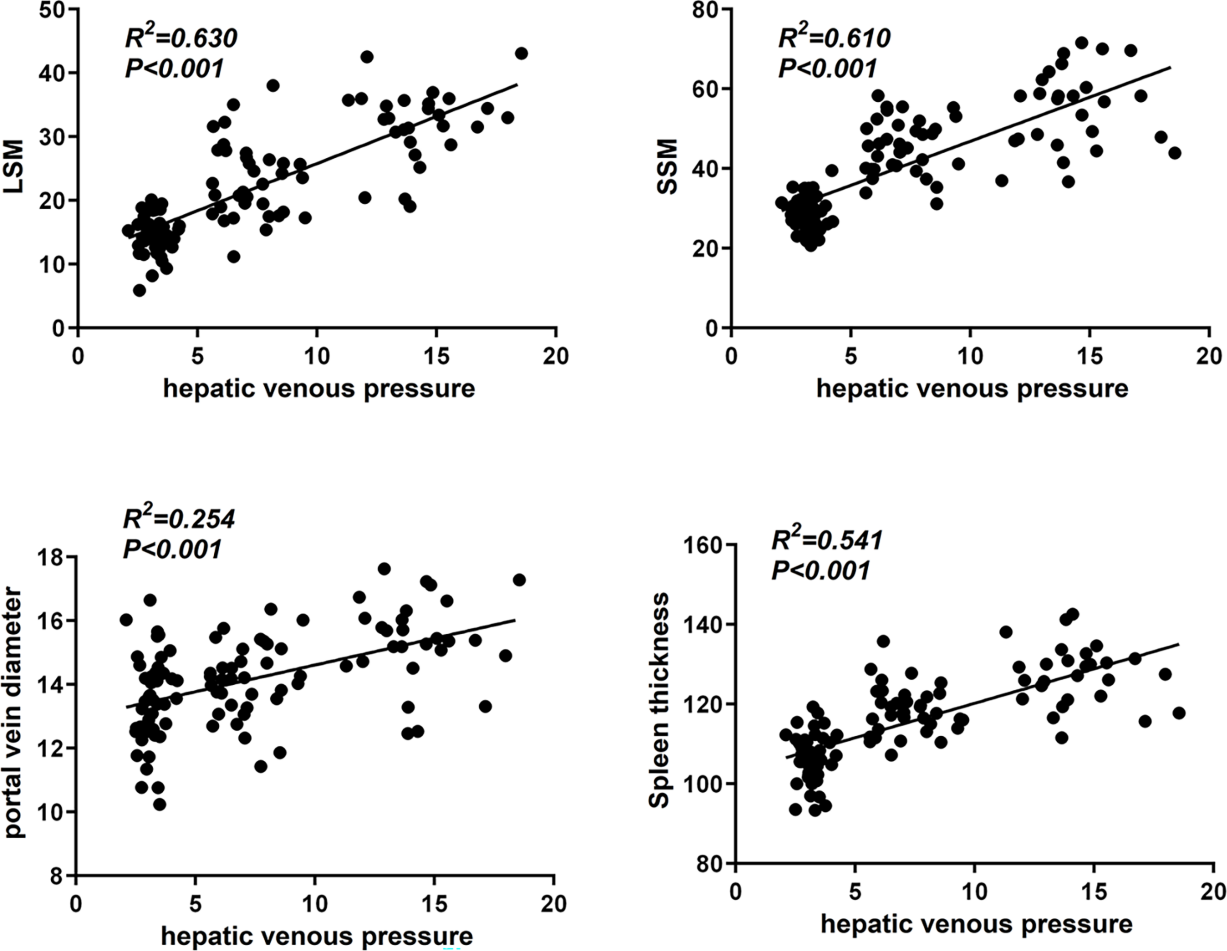
Correlation analysis of LSM, SSM, portal vein diameter and spleen thickness with hepatic vein pressure.

LSM, SSM, portal vein diameter and spleen thickness were positively correlated with hepatic vein pressure. These results indicate that LSM, SSM, portal vein diameter and spleen thickness may reflect hepatic venous pressure to some extent (all *P* < 0.05, Fig. 4) (Pearson correlation analysis).

### Diagnostic value of LSM, SSM, portal vein diameter and spleen thickness for clinically significant portal hypertension

**Figure 5:**
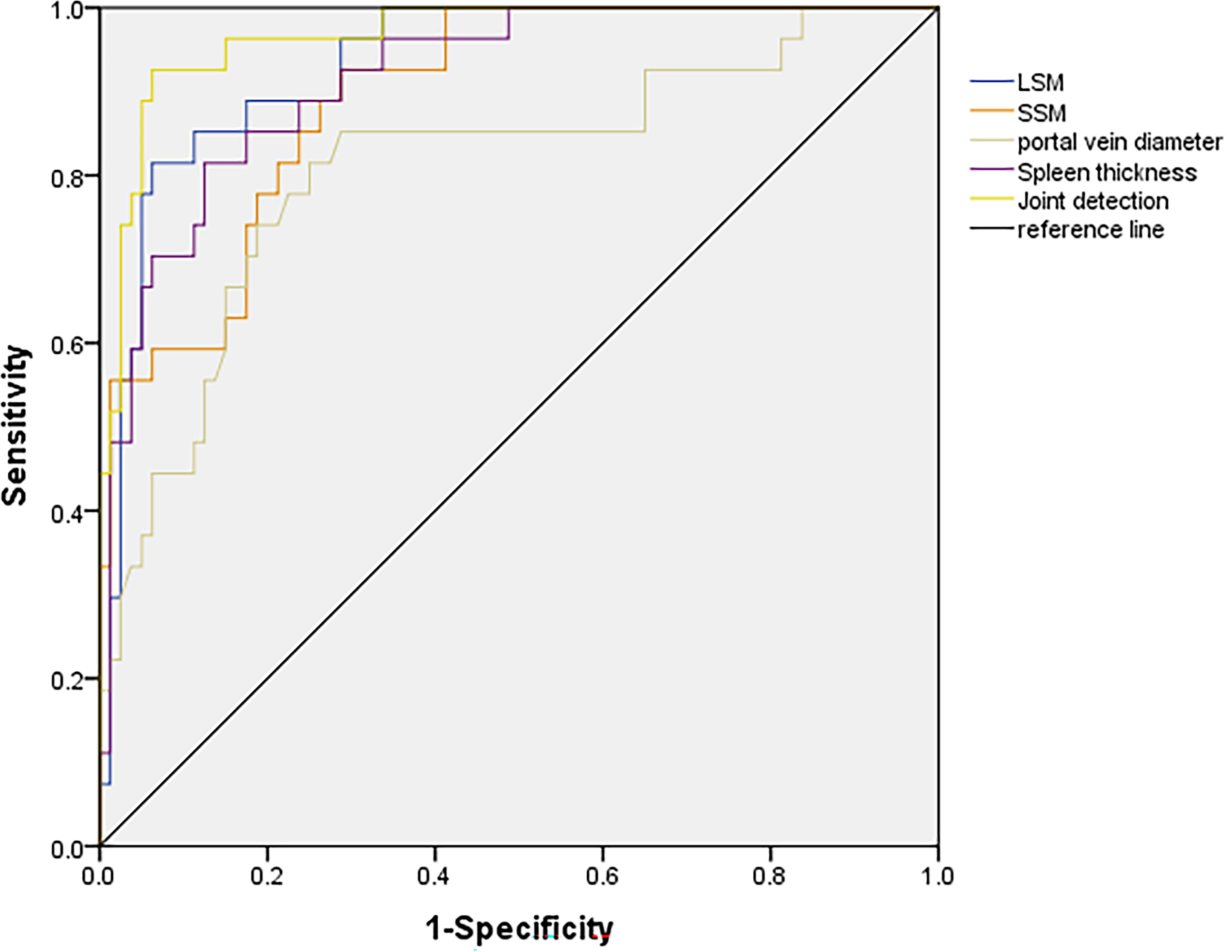
Diagnostic value analysis of LSM, SSM, portal vein diameter and spleen thickness for clinically significant portal hypertension.

ROC curve showed that AUC of combined detection was greater than that of SSM, portal diameter and spleen thickness detection alone. The results indicate that the combined detection of LSM, SSM, portal diameter and spleen thickness can be used to diagnose clinically significant portal hypertension (all *P* < 0.05, Table 3 and Fig. 5) (ROC test).

**Table 3:** Diagnostic value of LSM, SSM, portal vein diameter and spleen thickness for cirrhosis.

Indicator	Cutoff value	AUC	SE	95% CI
LSM	27.97kPa	0.933	0.025	0.885∼0.981
SSM	41.14kPa	0.895^*^	0.031	0.834∼0.957
Portal vein diameter	14.54cm	0.810^*^	0.052	0.708∼0.911
Spleen thickness	120.58cm	0.915^*^	0.029	0.859∼0.972
Combination		0.966	0.017	0.934∼0.999

**Notes.**

Note: Compared with combination, * *P* < 0.05.

### Logistic regression analysis of LSM, SSM, portal vein diameter and spleen thickness with increased hepatic vein pressure

The increase of LSM, SSM, portal vein diameter and spleen thickness were the influencing factors for the increase of hepatic vein pressure. It was further proved that these indicators were related to hepatic venous pressure (all *P* < 0.05, Table 4) (multivariate logistic regression analysis).

**Table 4:** Diagnostic value analysis of LSM, SSM, portal vein diameter and spleen thickness for clinically significant portal hypertension.

Indicator	*β*	SE	wald *χ*^2^	OR	95% CI	*P*-value
LSM	0.111	0.068	2.665	1.117	0.978∼1.277	0.103
SSM	0.131	0.054	5.885	1.140	1.025∼1.267	0.016
Portal vein diameter	0.518	0.248	4.363	1.679	1.032∼2.729	0.037
Spleen thickness	0.156	0.058	7.234	1.169	1.043∼1.310	0.007
Constant	−3.671	1.035	12.580	0.025	0.003∼0.194	<0.001

**Notes.**

Assignment: LSM (≥27.97 kPa = 1, <27.97 kPa = 0); SSM (≥41.14 kPa = 1, <41.14 kPa = 0); Portal vein diameter (≥ 14.54 cm = 1, <14.54 cm = 0); Spleen thickness (≥ 120.58 cm = 1, <120.58 cm = 0).

## Discussion

Portal hypertension is the main cause of esophageal and gastric varices rupture and bleeding and even death in patients with cirrhosis. According to clinical data, portal hypertension is a common complication in patients with cirrhosis, which can cause ascites, varicose esophagogastric fundus and splenomegaly, and can lead to death in severe cases (Ding et al., 2022). It has also been reported that the degree of portal hypertension is closely linked to the severity of liver disease both functionally and histologically (Xing et al., 2020; García-Pagán et al., 2020). As the gold standard for the diagnosis of portal hypertension, HVPG can reflect the severity of the disease and the prognosis of patients. However, it is an invasive examination with high requirements on medical conditions, professional level of operators and postoperative patient care (Benz et al., 2020). Ultrasonic shear wave elastography is a non-invasive ultrasonic quantitative evaluation technology. By generating shear waves that can propagate in human tissues and receiving reflected echoes, this technology can detect the propagation speed of shear waves in tissues to reflect the hardness of tissues. The greater the hardness of tissues, the faster the propagation speed of shear waves is. For another thing, compared with conventional ultrasound elastography, two-dimensional shear wave elastography is less susceptible to obesity, ascites and other factors, and can better reflect the real situation of tissue elasticity. Moreover, it makes up for the shortcomings of acoustic radiation force pulse imaging, which can only detect tissue elasticity near the focal point due to limited sampling area (Mărginean et al., 2020; Fofiu et al., 2021). Relevant reports indicate that two-dimensional shear wave elastography can mark the elasticity of different tissues in different colors to reflect the hardness of liver and spleen (Liu et al., 2022). The results of the present study showed that LSM and SSM values, portal vein diameter and spleen thickness in the cirrhosis group were higher than those in the hepatitis group, indicating that LSM, SSM and portal vein diameter and spleen thickness were increased in the cirrhosis patients. This was mainly because the spleen position was close to the body surface, and shear wave elastic imaging could obtain reliable spleen hardness values. As the splenic vein and portal vein continue behind the pancreatic neck, the splenic vein blood flow will be blocked when portal vein hypertension occurs. This leads to changes in the spleen, such as sinus congestion and parenchymal fibrosis, and further changes in the morphology of liver and spleen (Yan et al., 2021). In addition, this study found that the AUC of SSM, portal vein diameter and spleen thickness combined detection in the diagnosis of cirrhosis was greater than 0.9, indicating that the combined detection of all indicators has differentially diagnostic value for cirrhosis and hepatitis, and it may be applied in the early diagnosis of cirrhosis.

As portal hypertension is closely related to the severity of liver disease, the detection of portal venous pressure is of vital importance for evaluating the severity and prognosis of cirrhosis patients (Fanrong & Huadong, 2021; Zhang et al., 2021). Relevant studies indicate that portal hypertension not only causes splenomegaly, but also changes in splenic blood flow, tissue hyperplasia and fibrosis (Deng et al., 2019; Galina et al., 2021). The hardness of liver and spleen can reflect the degree of liver lesions to a certain extent, and liver portal hypertension is closely related to the degree of cirrhosis, suggesting that liver and spleen hardness may be related to liver portal hypertension (Long et al., 2022; Thiele et al., 2020). As the present study showed, LSM, SSM, portal vein diameter and spleen thickness were positively correlated with hepatic vein pressure. Further analysis found the rising LSM, SSM, portal vein diameter and spleen thickness to be the influencing factors of hepatic vein pressure increase. The increase of liver and spleen stiffness and the thickening of spleen could lead to the increase of hepatic vein pressure. This is mainly because in the process of liver fibrosis, liver stiffness increases and intrahepatic vascular resistance increases, resulting in increased hepatic venous pressure (Ye et al., 2020; Trebicka et al., 2022; Wu et al., 2020). In our study, the AUC of the combined detection of AUC, SSM, portal diameter and spleen thickness of clinically significant portal hypertension was greater that of measured separately, showing diagnostic value for portal hypertension. The higher the hepatic vein pressure, the greater the hardness of liver and spleen, because with the progression of cirrhosis, the viscera enter a high circulation state, and the congestion of liver and spleen is aggravated. In addition, the establishment of collateral circulation of portal body changes the hemodynamics of liver and spleen, leading to the increase of hardness of liver and spleen (Enliang et al., 2020). As the splenic vein and portal vein are extended behind the pancreatic neck, the splenic vein blood flow will be blocked when portal vein hypertension occurs, resulting in the splenic sinus congestion and expansion and therefore the change of spleen thickness (Zhipeng, Siliang & Jianbo, 2019; Shimeng et al., 2020). This study aimed to explore the relationship between liver and spleen hardness and portal vein pressure measured by two-dimensional shear wave elastography, which has certain novelty. However, the limited case number may lead to biased results, so studies of larger sample size are needed for further exploration.

## Conclusions

LSM and SSM in patients with compensatory viral cirrhosis is positively correlated with hepatic vein pressure, and the combined detection of LSM and SSM has diagnostic and predictive value for changes in portal vein pressure.

## Supplemental Information

10.7717/peerj.15956/supp-1 Supplemental Information 1 Raw data.

## Additional Information and Declarations

### Data Deposition

The following information was supplied regarding data availability: The raw data is available in the Supplemental File.
